# Clinical relevance between sodium-glucose co-transporter 2 inhibitors and lipid profiles in Asian patients with type 2 diabetes mellitus: a systematic review with a meta-analysis of randomized controlled trials

**DOI:** 10.1186/s40780-020-00160-0

**Published:** 2020-03-14

**Authors:** Junichi Mukai, Ayano Yoshiyama, Rie Kubota

**Affiliations:** Division of Clinical Pharmacy (Laboratory of Clinical Pharmacy Education) and Research and Education Center for Clinical Pharmacy, School of Pharmacy, Kitasato University, 5-9-1 Shirokane, Minato-ku, Tokyo, 108-8641 Japan

**Keywords:** Meta-analysis, Systematic review, Sodium-glucose co-transporter 2 inhibitors, Lipid profiles, Asian descent, Type 2 diabetes mellitus

## Abstract

**Background:**

Few systematic reviews have examined the effects of sodium-glucose co-transporter 2 inhibitors (SGLT2is) on lipid profiles in Asian patients with type 2 diabetes mellitus. We conducted a systematic review with a meta-analysis to summarize the available literature and confirm the effects of SGLT2is on lipid profiles in these patients.

**Methods:**

We searched the electronic databases MEDLINE, CENTRAL, and Ichushi-web for studies from the dates of their earliest publication to July 2018, and there was no language restriction. Trials were included if they were randomized controlled trials (RCTs) (1) comparing the effects of SGLT2is with a placebo in Asian patients with type 2 diabetes mellitus (18 years or older), and (2) reporting HbA1c and at least one lipid parameter, such as triglycerides (TG), high-density lipoprotein cholesterol (HDL-C), or low-density lipoprotein cholesterol (LDL-C). The weighted mean difference with a 95% confidence interval (CI) was calculated using a random-effects model.

**Results:**

Among the 630 studies retrieved, 17 RCTs that included 4485 patients were ultimately included in our review. Fourteen RCTs were conducted in Japan. The durations of RCTs ranged between 12 and 24 weeks. SGLT2is significantly improved HbA1c [mean difference − 0.80 (95%CI − 0.96 to − 0.64)%, *p* < 0.00001], TG [mean difference − 16.42 (95%CI − 22.71 to − 10.12) mg/dL, *p* < 0.00001], and HDL-C [mean difference 3.36 (95%CI 2.73 to 3.98) mg/dL, *p* < 0.00001], but significantly deteriorated LDL-C [mean difference 3.00 (95%CI 1.18 to 4.82) mg/dL, *p* < 0.001]. The LDL-C/HDL-C ratio was not significantly different between SGLT2is and a placebo [mean difference − 0.01 (95%CI − 0.08 to 0.06), *p* < 0.74].

**Conclusion:**

The present results suggest that in Asian patients with type 2 diabetes mellitus, TG and HDL-C values were better, while LDL-C values were worse with SGLT2is than with a placebo. However, the negative impact of SGLT2is on lipid profiles was modest. Further RCTs with a longer duration or conducted in other Asian countries are needed to provide further evidence to support the clinical relevance of changes in lipid profiles. The present results will be informative for SGLT2is users with concerns regarding the effects of SGLT2is on lipid profiles.

## Background

Sodium-glucose co-transporter 2 inhibitors (SGLT2is) are a new class of oral hypoglycemic agents that exert effects on glycemic control and weight loss [1]. The EMPA-REG OUTCOME study [2], which included patients with type 2 diabetes mellitus at a high risk of cardiovascular events, recently demonstrated that empagliflozin lowered the risk of death from cardiovascular events. The CANVAS program also showed that patients treated with canagliflozin had a lower risk of cardiovascular events [3]. As elevated levels of low-density lipoprotein cholesterol (LDL-C) are a well-established risk factor for cardiovascular disease [4, 5], and SGLT2is may reduce these levels. However, there are two conflicting studies on the effects of SGLT2is. The American Diabetes Association guidelines reported that SGLT2is had a negative impact on LDL-C [6], while an RCT conducted in Japan showed the opposite effects [7]. Racial differences generally exist between Asians and non-Asians. For example, Asians are more likely to have a lower body mass index than those of European descent [8]. They also have a higher percent body fat than Caucasians with the same body mass index [9]. Regarding lipid profiles, a recent study by Zhang and colleagues [10] indicated that the effects of metformin on high-density lipoprotein cholesterol (HDL-C) varied between ethnic groups. Based on these findings, we hypothesized that the effects of SGLT2is on lipid profiles differed between Asian patients and those of European descent.

To the best of our knowledge, few systematic reviews have examined the effects of SGLT2is on lipid profiles in Asian patients with type 2 diabetes mellitus. A systematic review by Cai and colleagues [11] investigated the effects of SGLT2is in these patients; however, the findings obtained need to be interpreted with caution because the term “Asian patients” used in their meta-analysis indicates that there are 50% or more Asian patients in each RCT selected. We herein conducted a systematic review with a meta-analysis to summarize the available literature and evaluate the clinical relevance between SGLT2is and lipid profiles in Asian patients with type 2 diabetes mellitus.

## Methods

### Searching strategies to identify randomized controlled trials (RCTs)

We searched the electronic databases MEDLINE, The Cochrane Central Register of Controlled Trials (CENTRAL), and Japana Centra Revuo Medicina (Ichushi-web) for studies from the dates of their earliest publication to July 2018. We included nine types of SGLT2is: canagliflozin (CANA), dapagliflozin (DAPA), empagliflozin (EMPA), ertugliflozin, ipragliflozin (IPRA), luseogliflozin (LUSEO), remogliflozin, sergliflozin, and tofogliflozin (TOFO). We used individual SGLT2i names, alternative names, “sodium-glucose transporter 2”, and “SGLT2 inhibitors” as search terms. We restricted our search to “randomized controlled trial” in these electronic databases. A reference search was also implemented from relevant studies in order to identify more RCTs. We did not impose any language restriction. Trials were included if they were RCTs (1) comparing the effects of SGLT2is with a placebo in Asian patients with type 2 diabetes mellitus (18 years or older), and (2) reporting HbA1c and at least one lipid parameter, such as triglycerides (TG), HDL-C, or LDL-C. We excluded cross-over trials and RCTs involving healthy subjects. The study search was undertaken independently by two authors (AY and JM). Any discrepancies were settled by discussions between the two assessors. We extracted data on the trial country, trial design, comorbidities, co-interventions, daily dose of each SGLT2i, duration of the intervention, and lipid profiles: TG, HDL-C, and LDL-C, at baseline. Lipid profiles were set as the primary endpoint and the LDL-C/HDL-C ratio as the secondary endpoint. In order to convert mmol/L of TG, HDL-C, and LDL-C to mg/dL, we multiplied mmol/L by 88.6, 38.7, and 38.7 respectively. Our systematic review with a meta-analysis did not require Ethics Committee approval.

### Quality assessment of each RCT

Study quality was quantified by both the Jadad scale and risk of bias tool. The Jadad scale is used to evaluate the appropriateness of the randomization technique, the method used for double-masking, and descriptions of dropouts or withdrawals [12]. The scale ranges between zero and five. We included studies that scored 4 points or higher in the analysis. The risk of bias for the studies was assessed based on the Cochrane Handbook [13]. Seven items were examined for the risk of bias: random sequence generation, allocation concealment, the blinding of participants and personnel, blinding of outcome assessments, incomplete outcome data, free of selective reporting, and a baseline imbalance for lipid parameters as other sources of bias. Each of the seven items was scored as a “low risk”, “unclear risk”, or “high risk”.

### Statistical analysis

We calculated the weighted mean difference with a 95% confidence interval (CI) for each outcome. The heterogeneity of each outcome was evaluated using chi-squared and I^2^ statistics. A value of 50% or more was defined to represent marked heterogeneity based on the Cochrane handbook [13]. We used a random-effects model (the DerSimonian and Laird method [14]) to assess outcomes more conservatively. In the meta-analysis, multiple SGLT2i groups in a single trial were combined into a single group [13]. Subgroup analyses were performed by including only Japanese patients and only patients who were treated with SGLT2is as monotherapy. We used Egger’s regression test [15] to assess publication bias more precisely when there were 10 RCTs or more in the meta-analysis [13]. All statistical analyses were performed with SPSS version 23.0 (SPSS Japan Inc., Tokyo, JAPAN) and review manager 5.3 software (Cochrane Collaboration, Oxford, UK). A *P* value less than 0.05 was considered to be significant.

## Results

**Fig. 1:**
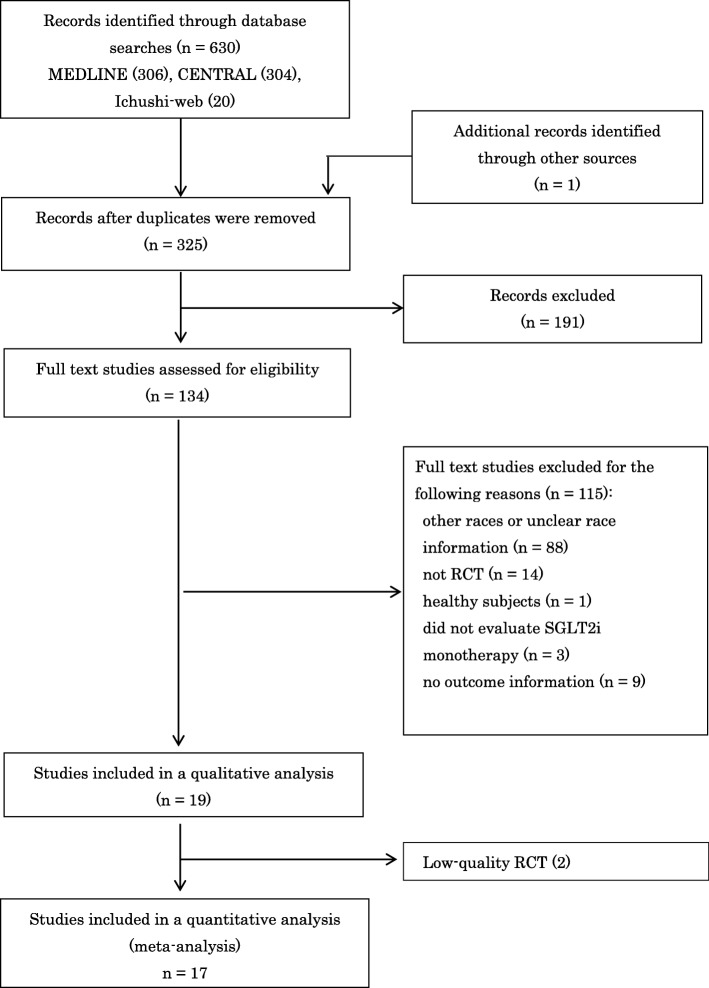
Identification process for eligible RCTs following PRISMA. Abbreviations: SGLT2i, sodium-glucose co-transporter 2 inhibitor; RCT, randomized controlled trial.

We identified 630 studies in the database search. One hundred and thirty-four full texts were retrieved after screening titles and abstracts. Seventeen RCTs that include 4485 patients were ultimately included in our review. Figure 1 shows the identification process for eligible RCTs [16–32] following PRISMA [33]. Table 1 shows the characteristics of RCTs included in the meta-analysis. All trials were published in English. Six types of SGLT2is (CANA, DAPA, EMPA, IPRA, LUSEO, and TOFO) were collected. Fourteen studies were conducted in Japan. The durations of RCTs ranged between 12 and 24 weeks.

**Table 1:** Characteristics of 17 randomized, double-blind, controlled trials included in the meta-analysis

Author	Country	Comorbid, co-intervention	Doses [mg/day], (n)	Duration (weeks)	HbA1c at baseline (%)	TG at baseline (mg/dL)	HDL-C at baseline (mg/dL)	LDL-C at baseline (mg/dL)	Jadad Scale
Ji 2014 [16]	China, Korea, Taiwan India	Diet and exercise	DAPA: 5 (128), 10 (133), P: (132)	24	DAPA: 8.1, 8.3, P: 8.4	NR	NR	NR	5
Kadowaki 2014 [17]	Japan	Diet and exercise	EMPA: 5 (110), 10 (109), 25 (109), 50 (110), P: (109)	12	EMPA: 7.9, 7.9, 7.9, 8.0, P: 7.9	EMPA:148.8, 128.5, 146.2, 148.8, P: 144.4	EMPA: 55.3, 58.8 57.7, 57.7, P: 57.3	EMPA: 127.3, 125.0, 125.0, 123.8, P: 124.2	4
Kashiwagi 2015A [18]	Japan	Renal impairment, diet/exercise, or using an OHA	IPRA: 50 (118), P: (46)	24	IPRA: 7.5, P: 7.5	IPRA: 137.6, P: 123.4	IPRA: 57.0, P: 56.4	IPRA: 114.3, P: 112.4	5
Lu 2016 [19]	Korea, Taiwan	Diet, exercise, and metformin	IPRA: 50 (87), P: (83)	24	IPRA: 7.7, P: 7.8	NR	NR	NR	5
Kashiwagi 2015B [20]	Japan	Diet and metformin	IPRA: (112), P: (56)	24	IPRA: 8.3, P: 8.4	IPRA: 165.4, P: 129.3	IPRA: 53.6, P: 57.4,	IPRA: 108.0, P: 113.6	4
Kashiwagi 2015C [21]	Japan	Sulfonylurea	IPRA: 50 (165), P (75)	24	IPRA: 8.4, P: 8.3	IPRA: 159.6, P: 151.3	IPRA: 57.6, P: 58.4	IPRA: 124.2, P: 120.4	5
Kashiwagi 2015D [22]	Japan	Pioglitazone	IPRA: 50 (97), P: (54)	24	IPRA: 8.2, P: 8.4	IPRA: 142.9, P: 135.2	IPRA: 61.1, P: 61.3	IPRA: 116.7, P: 130.4	5
Kashiwagi 2015E [23]	Japan	Diet and exercise	IPRA: 50 (62), P: (67)	16	IPRA: 8.4, P: 8.3	IPRA: 159.4, P: 148.1	IPRA:56.0, P: 52.1	IPRA: 124.4, P: 127.1	5
Haneda 2016 [24]	Japan	Renal impairment, Diet/exercise or using 1–2 OHAs	LUSEO: 2.5–5.0 (95), P: (50)	24	LUSEO: 7.7, P: 7.7	LUSEO: 147.7, P: 148.1	LUSEO:57.7, P:52.9	LUSEO: 115.1, P: 119.3	4
Seino 2014A [25]	Japan	Diet	LUSEO: 2.5 (79), P: (79)	24	LUSEO: 8.1, P: 8.2	LUSEO: 149.5, P: 141.5	LUSEO: 58. 0, P: 60.2	LUSEO: 131.0, P: 127.8	5
Seino 2014B [26]	Japan	Diet	LUSEO: 1.0 (55), 2.5 (56), 5 (54), 10 (58), P: (57)	12	LUSEO: 7.8, 8.1, 7.9, 8.0, P: 7.9	LUSEO: 156.1, 167.6, 136.2, 124.7, P:165.7	LUSEO: 56.7, 53.6, 54.2, 58.7, P: 55.0	LUSEO: 126.1, 128.8, 115.4, 121.4, P: 117.9	5
Seino 2014C [27]	Japan	Diet	LUSEO: 0.5 (60), 2.5 (61), 5 (61), P: (54)	12	LUSEO: 8.2, 8.1, 8.2, P: 7.9	LUSEO: 173.7, 150.2, 160.4, P:170.0	NR	NR	5
Inagaki 2016 [28]	Japan	Diet, exercise, and insulin	CANA: 100 (76), P: (70)	16	CANA: 8.9, P: 8.9	CANA: 124.5, P: 144.0	CANA: 61.9, P: 57.6	CANA: 122.4, P: 121.9	5
Ji 2015 [29]	China, Malaysia, Vietnam	Metformin alone or metformin plus sulfonylurea	CANA: 100 (223), 300 (227), P (226)	18	CANA: 8.0, 8.0, P: 7.9	CANA: 163.7, 180.8, P: 169.1	CANA: 51.0, 48.8, P: 49.1	CANA: 104.3, 100.8, P: 98.3	4
Inagaki 2014 [30]	Japan	Diet and exercise	CANA: 100 (90), 200 (88), P: (93)	24	CANA: 8.0, 8.0, P: 8.0	CANA: 150.9, 148.9, P: 158.1	CANA: 54.9, 55.3, P: 55.8	CANA: 127.3, 120.1, P: 124.8	5
Inagaki 2013 [31]	Japan	Diet and exercise	CANA: 50 (82), 100 (74), 200 (76), 300 (75), P: (75)	12	CANA: 8.1, 8.1, 8.1, 8.2, P: 8.0	NR	NR	NR	5
Kaku 2014 [32]	Japan	Diet and exercise	TOFO: 10 (57), 20 (58), 40 (58), P: (56)	24	TOFO: 8.5, 8.3, 8.4, P: 8.4	NR	NR	NR	5

*CANA* canagliflozin, *DAPA* dapagliflozin, *EMPA* empagliflozin, *IPRA* ipragliflozin, *LUSEO* luseogliflozin, *TOFO* tofogliflozin, *OHA* oral hypoglycemic agent, *P* placebo, *HDL-C* high-density lipoprotein cholesterol, *LDL-C* low-density lipoprotein cholesterol, *TG* triglycerides, *NR* not reported

### Quality assessment of each RCT

**Fig. 2:**
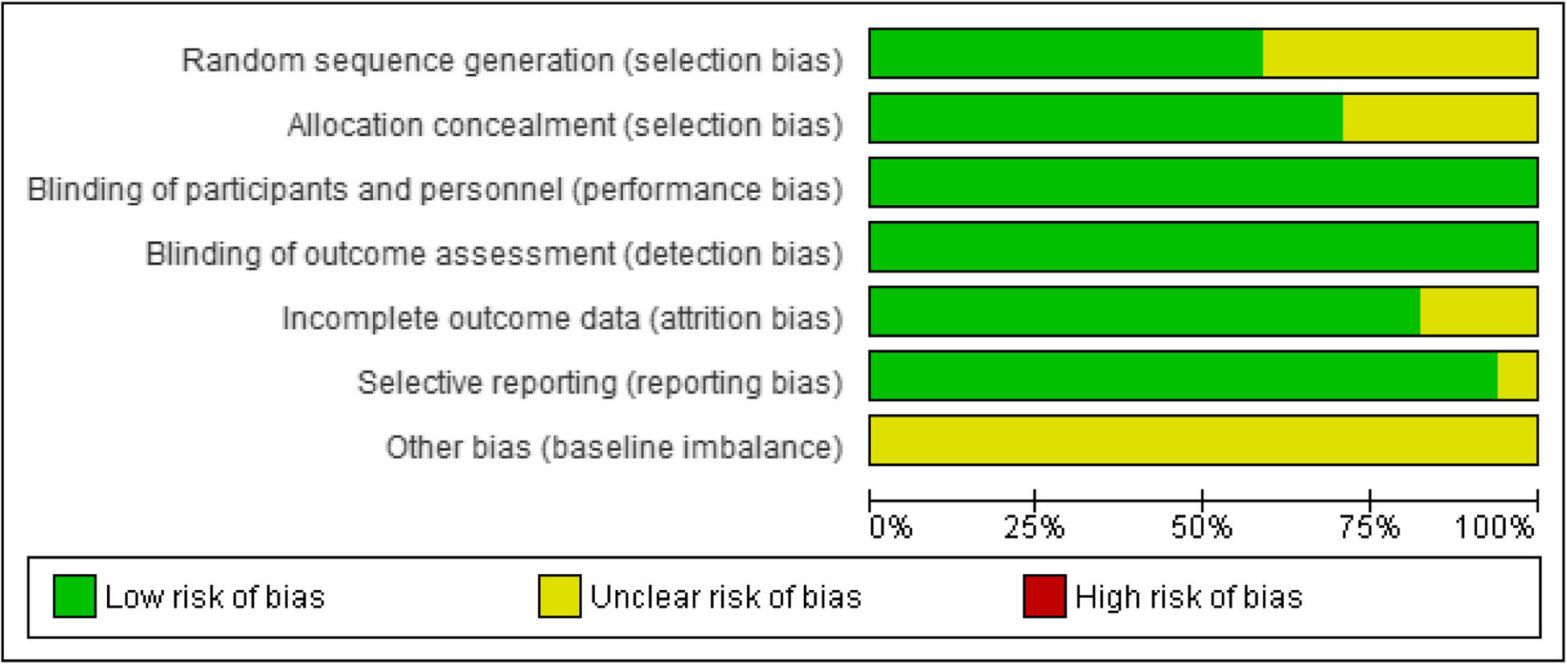
Risk of bias graph of 17 randomized controlled trials

The Jadad scale of the studies ranged between 4 and 5 points (Table 1). We also assessed the risk of bias of RCTs based on the Cochrane handbook [13]. Most studies were high-quality RCTs. “Low risk” was the highest in the domains of blinding of participants and personnel and blinding of outcome assessments. “Unclear risk” was the highest in the domain of baseline imbalance. “High risk” was not scored in all domains (Fig. 2). Egger’s regression test showed no significant results in all primary results.

### Relationship between SGLT2is and changes in HbA1

**Fig. 3:**
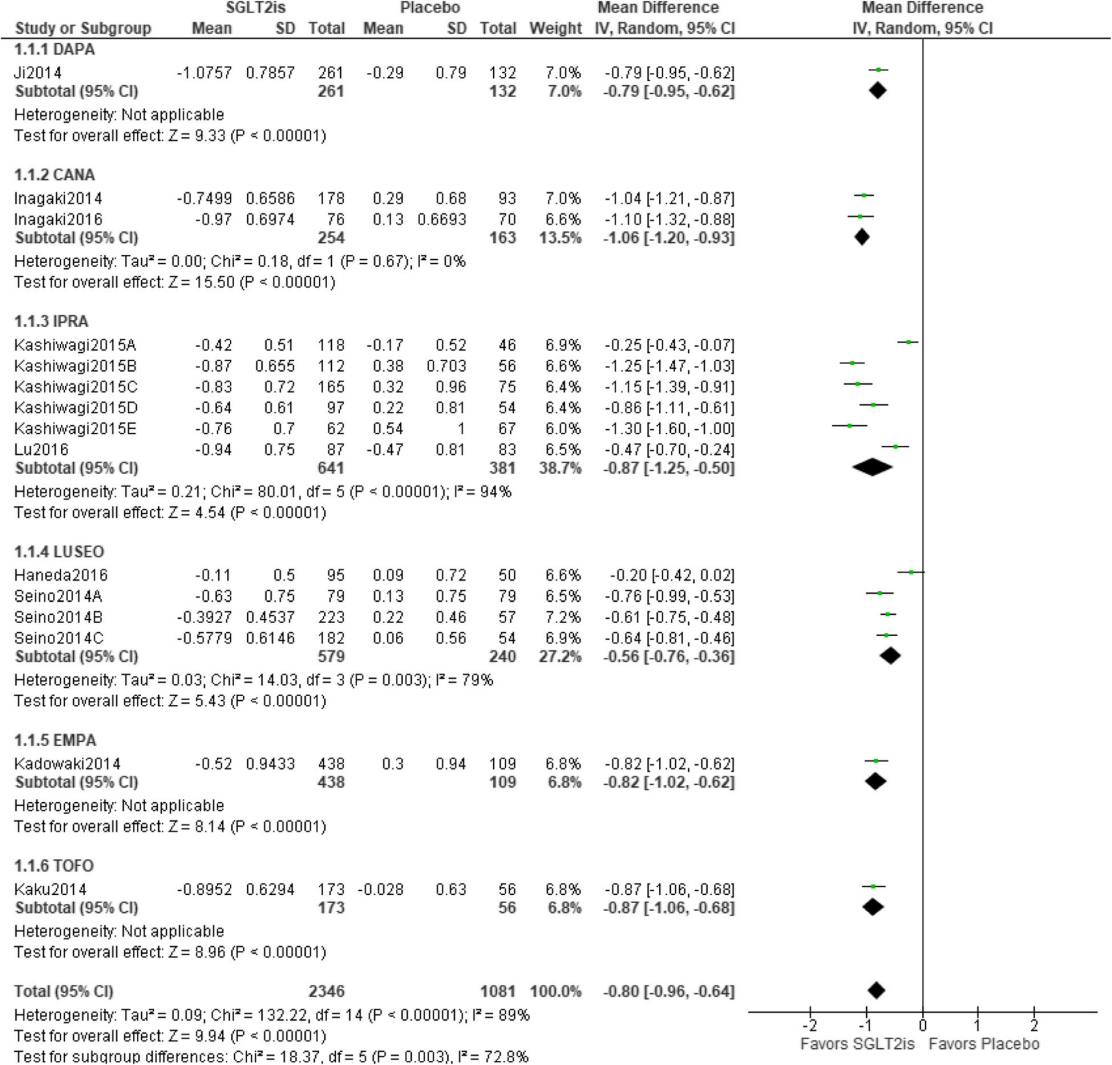
Relationship between SGLT2is and changes in HbA1c. Abbreviations: CANA, canagliflozin; DAPA, dapagliflozin; EMPA, empagliflozin; IPRA, ipragliflozin; LUSEO, luseogliflozin; TOFO, tofogliflozin; SGLT2i, sodium-glucose co-transporter 2 inhibitor; CI, confidence interval; SD, standard deviation.

Fifteen trials were included in the meta-analysis. Statistical heterogeneity was observed among trials (I^2^ = 89%). HbA1c values were significantly better with SGLT2is than with a placebo [mean difference − 0.80 (95%CI − 0.96 to − 0.64) %, *p* < 0.00001], and all types of SGLT2is showed a significant result in the sub-group analysis. The IPRA group had the highest weight (38.7%), whereas the EMPA and TOFO groups had the lowest weight (6.8% each) (Fig. 3).

### Relationship between SGLT2is and changes in TG

**Fig. 4:**
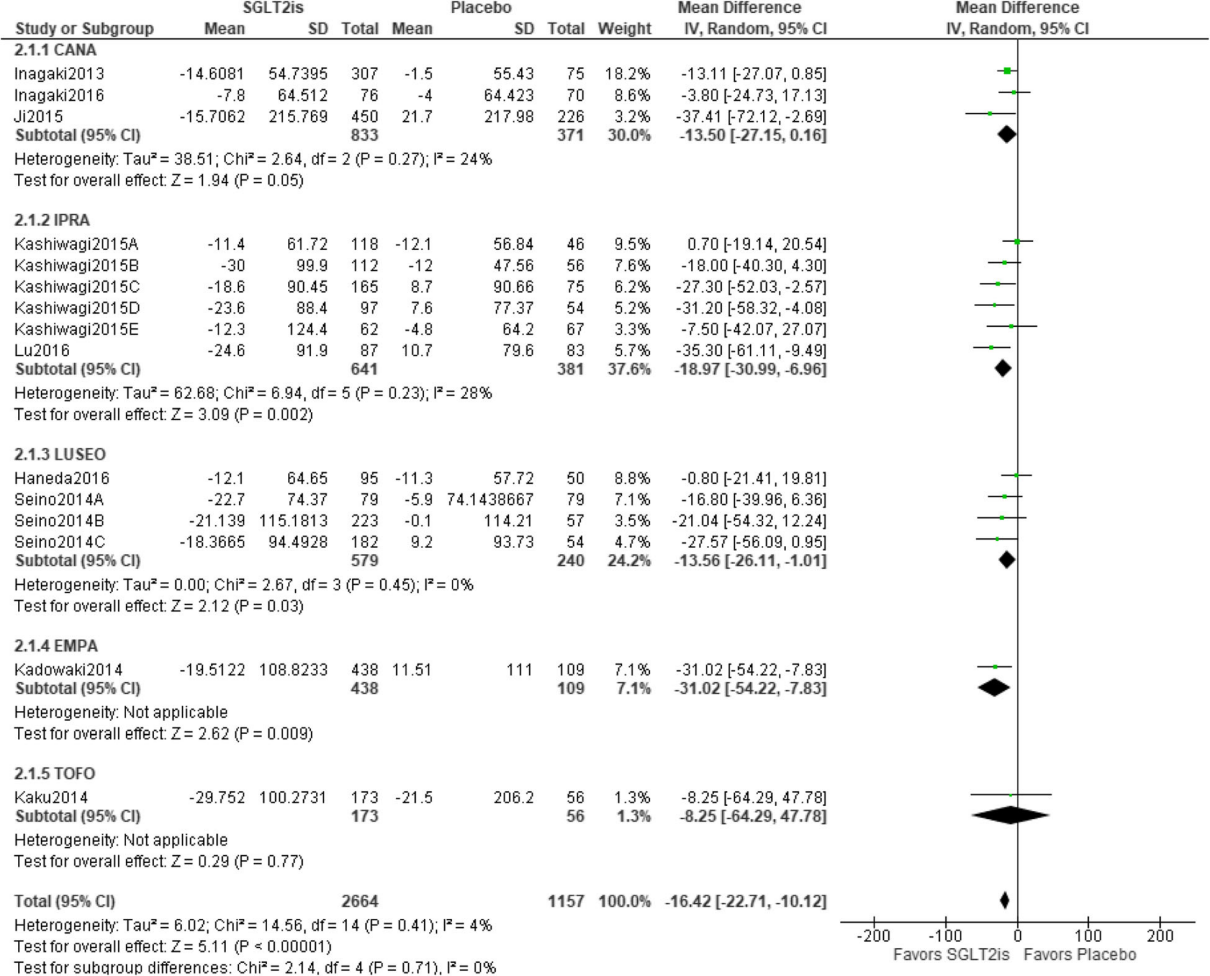
Relationship between SGLT2is and changes in TG. Abbreviations: CANA, canagliflozin; EMPA, empagliflozin; IPRA, ipragliflozin; LUSEO, luseogliflozin; TOFO, tofogliflozin; SGLT2i, sodium-glucose co-transporter 2 inhibitor; TG, triglycerides; CI, confidence interval; SD, standard deviation.

Fifteen trials were included in the meta-analysis. Statistical homogeneity was observed among trials (I^2^ = 4%). TG values were significantly better with SGLT2is than with a placebo [mean difference − 16.42 (95%CI − 22.71 to − 10.12) mg/dL, *p* < 0.00001], and the three types of SGLT2is examined (IPRA, LUSEO, and EMPA) showed a significant result in the sub-group analysis. The IPRA group had the greatest weight (37.6%), whereas the TOFO group had the lowest weight (1.3%) (Fig. 4).

### Relationship between SGLT2is and changes in HDL-C

**Fig. 5:**
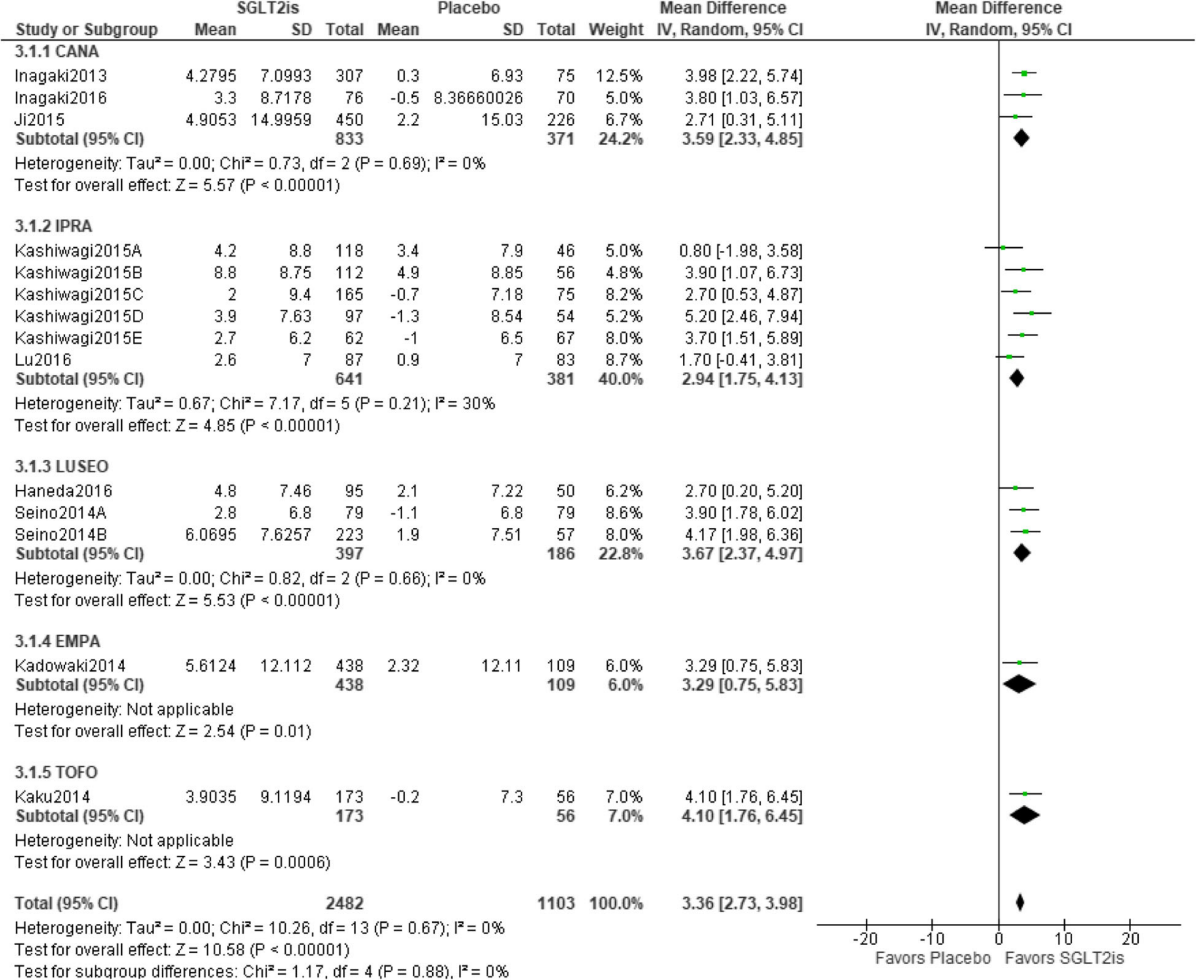
Relationship between SGLT2is and changes in HDL-C. Abbreviations: CANA, canagliflozin; EMPA, empagliflozin; IPRA, ipragliflozin; LUSEO, luseogliflozin; TOFO, tofogliflozin; SGLT2i, sodium-glucose co-transporter 2 inhibitor; HDL-C, high-density lipoprotein cholesterol; CI, confidence interval; SD, standard deviation.

Fourteen trials were included in the meta-analysis. Statistical homogeneity was observed among trials (I^2^ = 0%). HDL-C values were significantly better with SGLT2is than with a placebo [mean difference 3.36 (95%CI 2.73 to 3.98) mg/dL, *p* < 0.00001], and all types of SGLT2is showed a significant result in the sub-group analysis. IPRA group had the greatest weight (40.0%), whereas EMPA group had the lowest weight (6.0%) (Fig. 5).

### Relationship between SGLT2is and changes in LDL-C

**Fig. 6:**
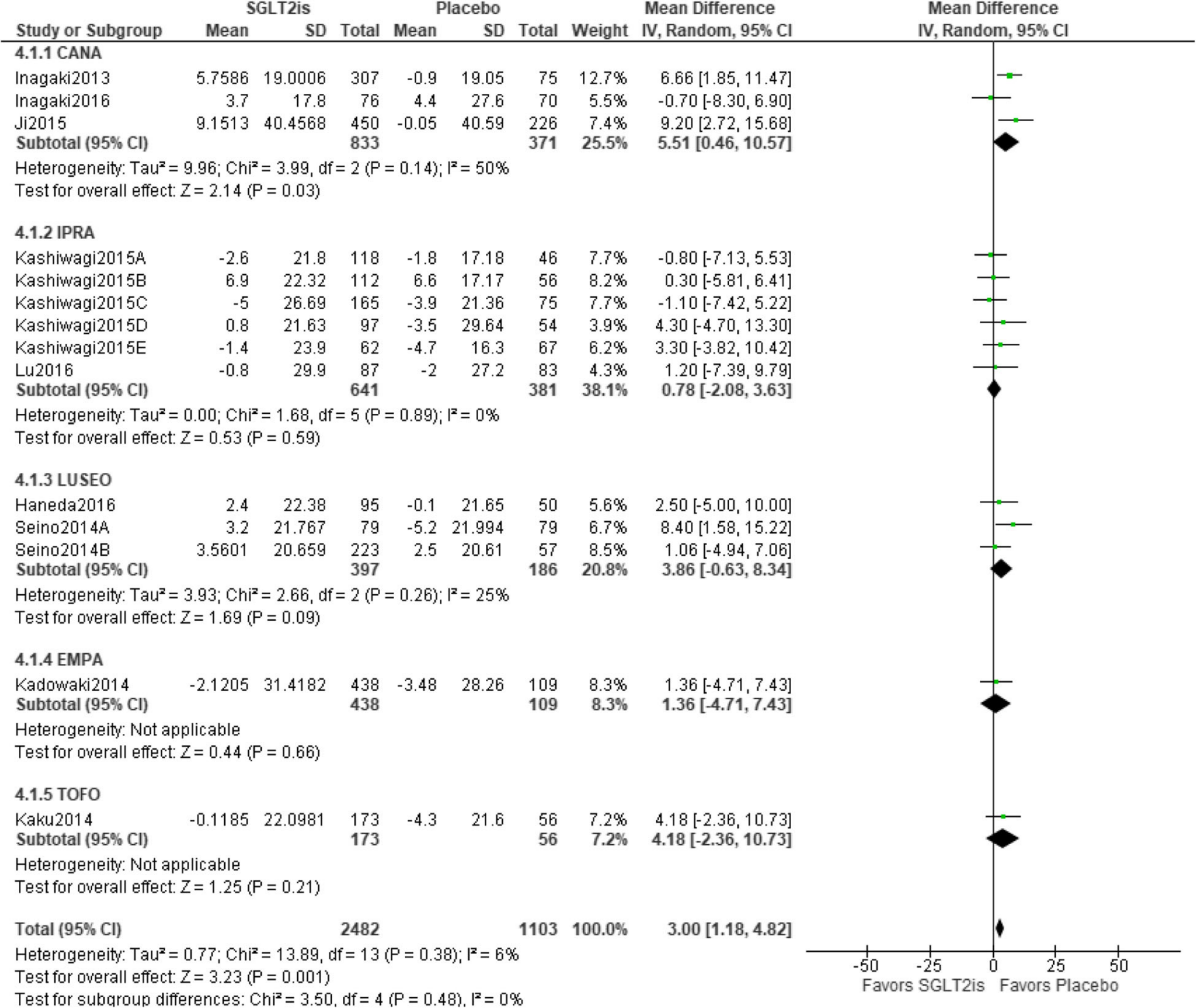
Relationship between SGLT2is and changes in LDL-C. Abbreviations: CANA, canagliflozin; EMPA, empagliflozin; IPRA, ipragliflozin; LUSEO, luseogliflozin; TOFO, tofogliflozin; SGLT2i, sodium-glucose co-transporter 2 inhibitor; LDL-C, low-density lipoprotein cholesterol; CI, confidence interval; SD, standard deviation.

Fourteen trials were included in the meta-analysis. Statistical homogeneity was observed among trials (I^2^ = 6%). LDL-C values were worse with SGLT2is than with a placebo [mean difference 3.00 (95%CI 1.18 to 4.82) mg/dL, *p* < 0.001], and only the CANA group showed a significant result in the sub-group analysis. The IPRA group had the greatest weight (38.1%), whereas the TOFO group had the lowest weight (7.2%) (Fig. 6).

### Relationship between SGLT2is and changes in the LDL-C/HDL-C ratio

**Fig. 7:**
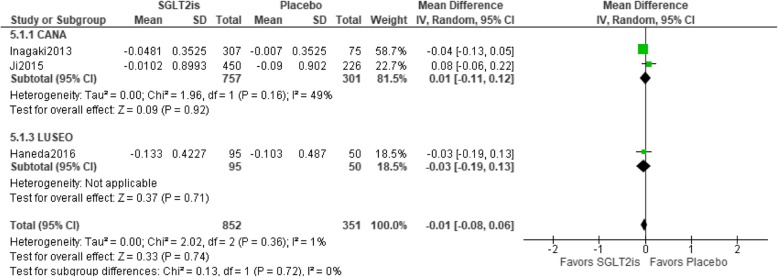
Relationship between SGLT2is and changes in the LDL-C/HDL-C ratio. Abbreviations: CANA, canagliflozin; LUSEO, luseogliflozin; SGLT2i, sodium-glucose co-transporter 2 inhibitor; HDL-C, high-density lipoprotein cholesterol; LDL-C, low-density lipoprotein cholesterol; CI, confidence interval; SD, standard deviation.

Three trials were included in the meta-analysis. Statistical homogeneity was observed among trials (I^2^ = 1%). The LDL-C/HDL-C ratio was not significantly different between SGLT2is and a placebo [mean difference − 0.01 (95%CI − 0.08 to 0.06), *p* < 0.74], and none of the groups showed a significant result in the sub-group analysis. The CANA group had the greatest weight (81.5%) (Fig. 7).

### Additional analyses

The results of the sub-group analysis including only Japanese patients and only patients who were treated with SGLT2is as monotherapy were consistent with the main results (Table 2).

**Table 2:** Summary of subgroup analyses

	Outcome	Trial, n	SGLT2i, n	Placebo, n	Mean difference [95%CI]	Heterogeneity (%)	Test for the overall effect (*p* value)
Only Japanese patients	HbA1c (%)	13	1998	866	− 0.83 [− 1.01, − 0.65]	90	< 0.00001
	TG (mg/dL)	13	2127	848	−14.39 [−20.80, −7.98]	0	< 0.0001
	HDL-C (mg/dL)	12	1945	794	3.58 [2.90, 4.25]	0	< 0.00001
	LDL-C (mg/dL)	12	1945	794	2.59 [0.72, 4.46]	0	0.007
	LDL-C/HDL-C ratio	2	402	125	−0.04 [−0.12, 0.04]	0	0.33
Only patients treated with SGLT2i as monotherapy	HbA1c (%)	8	1596	647	−0.84 [− 0.97, − 0.70]	77	< 0.00001
	TG (mg/dL)	7	1464	497	−17.96 [−27.03, −8.88]	0	0.0001
	HDL-C (mg/dL)	6	1282	443	3.89 [3.01, 4.76]	0	< 0.00001
	LDL-C (mg/dL)	6	1282	443	4.29 [1.81, 6.76]	0	0.0007
	LDL-C/HDL-C ratio	1	307	75	−0.04 [−0.13, 0.05]	NA	0.37

*SGLT2i* the sodium-glucose co-transporter 2 inhibitor, *HDL-C* high-density lipoprotein cholesterol, *LDL-C* low-density lipoprotein cholesterol, *TG* triglycerides, *CI* confidence interval, *NA* not applicable

## Discussion

We herein conducted a systematic review with a meta-analysis to summarize the available literature and confirm the effects of SGLT2is on lipid profiles in Asian patients with type 2 diabetes mellitus. The present study, which consisted of 17 RCTs including 4485 Asian patients with type 2 diabetes mellitus, suggests that TG and HDL-C values were better, whereas LDL-C values were worse with SGLT2is than with a placebo and also showed that there was no heterogeneity (I^2^ ≤ 6%) in each lipid profile.

Our results for lipid outcomes were consistent with the meta-analysis by Cai and colleagues [11]; a significant, but small change was observed in lipid outcomes, and these outcomes indicated high heterogeneity (I^2^ > 90%). This heterogeneity was attributed to their meta-analysis including RCTs with different inclusion criteria [11]. Total heterogeneity (I^2^ ≤ 6%) may also have been attributed to most of the SGLT2i subgroups having low heterogeneity in our analysis. Total heterogeneity was higher when we excluded the subgroup with low heterogeneity to confirm the impact of heterogeneity between SGLT2i groups in our meta-analysis. Incidentally, in our analysis, all SGLT2i groups with different doses in the treatment arm were combined into a single group based on the Cochrane Handbook [13]. In contrast, the study by Cai and colleagues [11] included only the standard dose of SGLT2is in each treatment arm; however, the impact of this methodological difference across meta-analyses currently remains unclear.

An increase of 1 mg/dL in HDL-C from baseline after 3 months may be expected to reduce the risk of major cardiovascular events by 1.1% in the post-hoc analysis of the TNT trial [34]. Similarly, all RCTs included showed consistent increases in HDL-C of 1 mg/dL or more from baseline after approximately 3 months before these RCTs were combined. HDL-C was 3.4 mg/dL higher with SGLT2is than with a placebo in our meta-analysis. This result suggests that SGLT2is exert protective effects against cardiovascular events in Asian populations. The present meta-analysis showed that SGLT2is decreased TG by 16.4 mg/dL and increased LDL-C by 3.0 mg/dL from placebo values. A recent meta-regression analysis with an average median trial duration of 4.8 years showed that the risk ratio of major vascular events was 0.92 per 40 mg/dL reduction in TG [35]. Another meta-analysis with a mean follow-up of 4.3 years [36] reported a 21% reduction in major vascular events per 1 mmol/L (38.7 mg/dL) reduction in LDL-C. Further RCTs with a longer duration are needed to establish whether the modest changes observed in TG and LDL-C in our meta-analysis are of clinical importance because the maximum duration of RCT in our review was too short (at most 24 weeks).

The RCT that included approximately 80% Caucasians also demonstrated that CANA at 300 mg increased TG by 20.2 mg/dL over the placebo value [37], while the results for TG in our meta-analysis showed the opposite effect [mean difference − 16.42 (95%CI − 22.71 to − 10.12) mg/dL]. The EMPA-REG OUTCOME study [2, 38], which included 72% Caucasians, and the RCT by Bode and colleagues [39], comprising 70% or more Caucasians, showed small increases in HDL-C and LDL-C over those with the placebo. The magnitude of effects on HDL-C and LDL-C were equal to the results of the present study including 100% Asian patients [mean difference 3.36 (95%CI 2.73 to 3.98) mg/dL, mean difference 3.00 (95%CI 1.18 to 4.82) mg/dL, respectively]. Therefore, the effects of SGLT2is on HDL-C and LDL-C do not appear to be dependent on race, although racial differences may explain this smaller increase in TG; however, differences in the types of SGLT2is used, such as CANA and EMPA, or patient backgrounds, which included those treated with antihyperlipidemic therapies or statins, may have affected this result [2, 37–39]. Further studies are needed to verify whether racial differences affect lipid metabolism.

Regardless of the types of SGLT2is, consistent results, such as a decrease in TG and increases in HDL-C and LDL-C, were observed in our meta-analysis. Similarly, weight loss in patients with type 2 diabetes mellitus in UKPDS reduced TG with an increase in HDL-C [40]. Weight loss with SGLT2is may explain, in part, the better TG and HDL-C values observed. Overall, there was no significant change in the LDL-C/HDL-C ratio in our analysis. This result supports the hypothesis that the increase in LDL-C induced by SGLT2is may be counterbalanced by elevated HDL-C [41]. A possible mechanism for the increase observed in LDL-C with SGLT2is is explained using a preclinical model. This increase may be due to the delayed clearance of LDL from the circulation along with elevated plasma lipoprotein lipase activity [42].

The present study has some limitations. Although Egger’s regression test showed no significant differences in primary outcomes, there may have been a publication bias because we only retrieved published studies. Furthermore, our review was unable to rule out the impact of other anti-hyperglycemic agents because it included some patients who were treated with an oral hypoglycemic agent or insulin as combination therapy. However, the results of the subgroup analysis that only included patients who were treated with SGLT2is as monotherapy were consistent with the main results (Table 2). Similarly, some lifestyle interventions, such as diet and exercise, which had been performed in most of the RCTs collected, may also have contributed to our lipid outcomes because these interventions are known to affect lipid profiles [43, 44]. The combination of lifestyle intervention(s) and oral hypoglycemic agent therapy is commonly used in clinical practice. In addition, the results obtained are hard to generalize for other Asian populations. The results of the sub-group analysis that included Japanese patients only were consistent with the main results. A possible reason for this is that Japanese patients accounted for 80% of the Asian population in this review. Another limitation is that the numbers of different types of SGLT2is that we pooled were unbalanced. There were also no lipid outcomes including all types of SGLT2is in our meta-analysis. The IRPA group with the highest weight may have affected all lipid profiles.

## Conclusion

In summary, the present results suggest that in Asian patients with type 2 diabetes, TG and HDL-C values were better, while LDL-C values were worse with SGLT2is than with a placebo. However, the negative impact of SGLT2is on lipid profiles was modest. Further RCT with a longer duration or conducted in other Asia countries are needed to provide further evidence to support the clinical relevance of changes in lipid profiles. The present results will be informative for SGLT2is users with concerns regarding the effects of SGLT2is on lipid profiles.

## Data Availability

The datasets used and/or analyzed during the present study are available from the corresponding author on reasonable request.

## Abbreviations

CANA: Canagliflozin
CI: Confidence interval
DAPA: Dapagliflozin
EMPA: Empagliflozin
HDL-C: High-density lipoprotein cholesterol
IPRA: Ipragliflozin
LDL-C: Low-density lipoprotein cholesterol
LUSEO: Luseogliflozin
RCT: Randomized controlled trial
SGLT2i: Sodium-glucose co-transporter 2 inhibitor
TG: Triglycerides
TOFO: Tofogliflozin
